# A anti-jamming method for satellite navigation system based on multi-objective optimization technique

**DOI:** 10.1371/journal.pone.0180893

**Published:** 2017-07-13

**Authors:** Rongling Lang, Hong Xiao, Zi Li, Lihong Yu

**Affiliations:** 1 School of Electronics and Information Engineering, Beihang University, Beijing, China; 2 Department of Foreign Language, Huaiyin Institute of Technology, Huai’an, Jiangsu, China; Nankai University, CHINA

## Abstract

In this paper, an anti-jamming method, which turns the single objective optimization problem into a multi-objective optimization problem by utilizing 2-norm, is proposed. The proposed jamming suppression method can reduce the wide nulls and wrong nulls problems, which are generated by the common adaptive nulling methods. Therefore a better signal-noise-ratio (SNR) can be achieved, especially when the jammers are close to satellite signals. It can also improve the robustness of the algorithm. The effectiveness of the proposed method is evaluated by simulation and practical outdoor experiments with the GPS L1 band C/A signals. The experimental results show that with the dedicated method, the nulls targeting at the corresponding jammers become narrower and the wrong nulls can be eliminated.

## 1. Introduction

The Global Navigation Satellite System (GNSS) has been an important component in both military and civilian fields, whereas it is easily interfered due to its own inherent defect. Taking the Global Position System (GPS) as an example, its satellites are up to 20000 kilometers away from the earth. This results in that the power of signal which is received by the ground receiver is very weak (about -160dBW), so GPS signal is susceptible to be jammed. The research on jamming and anti-jamming techniques for (GNSS) has also been widely studied.

The jammers affecting GNSS can be intentional and unintentional ones. Intentional jammers include deceiving jammers and suppressing jammers, of which the suppression jammers are dominant, and they suppress the navigation signals by emitting high power signals [1], such as broadband, narrowband or continuous wave (CW) [2–5]. Therefore, this paper focuses on the reduction of the suppressing jamming signals.

Generally, an antenna array for interference suppression is a spatial filter. It is a signal- processing method which makes use of the spatial selectivity of antenna array to filter out the jammers and to improve the SNR. The anti-jamming algorithms based on antenna array are divided into two categories according to their goals. One is the Digital Beam Forming (DBF) [6], which adjusts the main beam automatically to the desired signals. The other is the Adaptive Nulling algorithm [7], which points the nulls to jammers. The DBF requires the direction information of GNSS signals and its computation is quite complex. As a result, its jamming mitigation performance will degrade severely if errors of the estimation on GNSS signals’ direction increase. On the contrary, the Adaptive Nulling method, which is called a blind jamming suppression technique, does not need any prior information about the jammers. The Adaptive Nulling method will be investigated in details in this paper.

Since Adaptive Nulling method doesn’t use any transcendental information, the nulls may be too wide in the interference direction and there may be many ‘wrong nulls’ in the non-interfering direction. In order to solve the above problems, B.G. Agee proposed a Self-Coherence Recovery (SCORE) [8] algorithm based on GNSS signals’ property of periodic repetition. Without knowing the direction of GNSS signals, SCORE algorithm adds an auxiliary channel exactly after the main channel. It calculates the optimal weights through maximizing the cross correlation of signals between the main channel and the auxiliary channel under the Least Squire principle. This method not only can make the gains in some GNSS signal’s direction as high as possible, but also can form deep nulls in the direction of jammers automatically. However, this method is unable to guarantee that the main beam to aim at every GNSS signal. If the gains of the non-jamming direction remain unchanged, the above problems of Adaptive Nulling would be avoided. Wenyi Wang put forward a proposal that the gains in all directions should be close to 0 dB [9].

A new jamming suppression method for satellite navigation system based on multi-objective optimization algorithm of 2-norm is proposed to solve the above-mentioned problems in this paper. Furthermore, a mathematical model of spatial adaptive nulling with 2-norm constraint on uniform circular arrays is built as well. For theory verification, the Power Inversion (PI) algorithm [10–13] and Multiple Signal Classification (MUSIC) [14] are used as examples, based on which, the multi-objective optimization anti-jamming algorithms are deduced and the corresponding experiments are carried out and analyzed. The experimental results agree well with the proposed methods and prove our theory further.

The paper is organized as follows. In Section 2, the original PI and Music are introduced. In Section 3, the wide nulls and wrong nulls problems caused by the original algorithms are analyzed. The main contributions of the paper are presented in this section too, where a new jamming suppression criterion and its use in PI and MUSIC are proposed in details. In Section 4, experiments are carried out to verify our proposed method. Finally, the paper is concluded in Section 5.

## 2. The original PI and MUSIC

### 2.1 The model of signal received by antenna array

Supposing that an antenna array is made up of *M* units, accordingly the vector of received signals can be given as:

$$x=A_{\text{S}}S+A_{J}J+N$$ (1)

Where **x** = [*x*_1_, *x*_2_, ⋯, *x*_*M*_]^*T*^ is a column vector of received signals. *x*_*i*_ (*i* = 1, 2, ⋯, *M*) stands for the signal received by the *i*-th unit. **S** = [*s*_1_(*t*), *s*_2_(t), ⋯, *s*_*q*_(*t*)]^*T*^ is a column vector of *q* GNSS signals, and *s*_*l*_(*t*) (*l* = 1, 2, ⋯, *q*) is the *l-th* GNSS signal. **J** = [*j*_1_(*t*), *j*_2_(t), ⋯, *j*_*K*_(*t*)]^*T*^ is a column vector of *K* jammers, and *j*_*i*_(*t*) (*i* = 1, 2, ⋯, *K*) is the *i-th* jammer. **A**_*s*_ = [**α**_1_, **α**_2_, ⋯,**α**_*q*_]_*M*×*q*_ is the directional vector matrix of GNSS signals. $\alpha_{i}={\left[e^{j\frac{2\pi c}{\lambda_{0}}\nabla_{i1}},e^{j\frac{2\pi c}{\lambda_{0}}\nabla_{i2}},\cdots ,e^{j\frac{2\pi c}{\lambda_{0}}\nabla_{iM}}\right]}^{T}\;(i=1,2,\cdots q)$ is the directional vector of the signal *s*_*i*_(*t*), and ∇_*im*_ (*m* = 1, 2, ⋯, M) is the time delay when the signal *s*_*i*_(*t*) arrives at the *m-th* unit relative to the reference antenna. *c* is the transmission speed of light, and *λ*_0_ is the wavelength of the GNSS signal *s*_*i*_(*t*). **A**_*J*_ = [**β**_1_, **β**_2_, ⋯, **β**_*K*_]_*M*×*K*_ is the directional vector of jammers, and $\beta_{\tau }={\left[e^{j\frac{2\pi c}{\lambda_{0}}\nabla_{\tau 1}},e^{j\frac{2\pi c}{\lambda_{0}}\nabla_{\tau 2}},\cdots ,e^{j\frac{2\pi c}{\lambda_{0}}\nabla_{\tau M}}\right]}^{T}\;(\tau =1,2,\cdots K)$ means the directional vector of the jammer *j*_*τ*_(*t*). **N** = [*n*_1_, *n*_2_, ⋯, *n*_*M*_]^*T*^ is the vector of noise. *n*_*i*_ (*i* = 1, 2, ⋯, *M*) is the *i-th* antenna’s thermal noise, which is zero-mean Gauss distribution with variance equaling to *σ*^2^. These noises are independent on each other and uncorrelated to the GNSS signals.

### 2.2 The original PI

The spatial adaptive nulling technique adjusts the received signals with the weight vector **W** to eliminate jammers. The output signals is y = **W**^*H*^**X**, where **W** = [*w*_1_, *w*_2_…, *w*_*M*_]^*T*^ is the weight vector, and *w*_*m*_ (m = 1, 2,…M) is the *m-th* unit’s weight. *y* is the weighted sum of all the received signals.

The most important part of spatial adaptive nulling processing technique is how to calculate the optimal weight vector **W**_*opt*_. The regular criteria are Minimum Mean Square Error (MMSE), Maximum Signal to Interference and Noise Ratio (MSINR), Linearly Constrained Minimum Variance (LCMV) and subspace decomposition. We will take the LCMV and subspace decomposition as the examples in this paper.

LCMV is an optimal criterion which minimizes the output powers. Its optimization formula is written as:

$$\varepsilon (n)=\underset{w}{\text{min}}E[\left|y{\left(n\right)}^{2}\right|]=\underset{w}{\text{min}}E[w^{H}xx^{H}w]=\underset{w}{\text{min}}w^{H}Rw$$ (2)

where **R** = *E*[**xx**^*H*^] is correlation matrix, and is a positive definite matrix.

Obviously the minimum value of (Eq 2) is zero when **W** = **0**. Therefore, a common constraint is **w**^*H*^**h** = *d*, where **h** = [1 0 … 0]^*T*^ and *d* is an non-zero constant, generally taken as 1. Thereby the optimal model for spatial adaptive nulling can be written as:

$$\{\begin{array}{c} \varepsilon (\text{n})=\underset{w}{\text{min}}w^{H}Rw\\ w^{H}h=d \end{array}$$ (3)

PI is the most typical spatial adaptive processing algorithm based on LCMV.

### 2.2 The original MUSIC

Let *λ*_1_, *λ*_2_, ⋯ *λ*_*M*_ be the eigenvalues of **R**, then

$$\lambda_{1}\geq \lambda_{2}\geq \cdots \lambda_{K}>>\lambda_{K+1}=\lambda_{K+2}=\cdots \lambda_{M}=\sigma^{2}$$ (4)

Suppose **e**_1_, **e**_2_, ⋯ **e**_*K*_ are the eigenvectors of **R** corresponding to the first *K* bigger eigenvalues. **e**_1_, **e**_2_, ⋯ **e**_*K*_ span a subspace called jammer subspace. Suppose **e**_*K*+1_, **e**_2_, ⋯ **e**_*M*_ are the eigenvectors of **R** corresponding to the later *M* − *K* smaller eigenvalues, which are the base of the noise subspace. The jammer subspace is orthogonal to the noise subspace [14–16], namely

$$span\{e_{1},e_{2},\cdots e_{K}\}⊥span\{e_{K+1},e_{2},\cdots e_{M}\}$$ (5)

Since *span*{**e**_1_, **e**_2_, ⋯ **e**_*K*_} = *span*{**α**_1_, **α**_2_, ⋯ **α**_*q*_}, **W**_*opt*_ can be achieved in the noise subspace, namely $W_{opt}=\sum_{t=1}^{M-K}p_{t}e_{K+t}\in span\{e_{K+1},e_{2},\cdots e_{M}\}$, where *p*_*t*_ is the coefficient of **e**_*K*+*t*_. This is the basic idea of MUSIC used for anti-jamming [15, 16].

The PI algorithm and MUSIC will be taken as examples to evaluate the performance of the algorithm on the basis of 2-norm jamming suppression optimal principle.

## 3. The proposed algorithm

Jammer Reduction (JR) and Satellite Availability (SA) are the two crucial indexes when evaluating the performance of jamming suppression. Although PI algorithm is able to form nulls automatically in the interference direction without using the directional information of the GNSS signals and the jammers, the null in the interference direction may be so wide that the GNSS signals fall into the null, especially when jammers and GNSS signals are extremely close to each other. In this case, it is quite difficult for the GNSS receivers to acquire the GNSS signals.

For PI, Since $W_{opt}=\frac{R^{-1}h}{h^{T}R^{-1}h}$ and **h** = [1 0 … 0]^*T*^, if **R** has one or more small eigenvalues, the determinant of **R** is very small and **W**_*opt*_ maybe not been computed properly. Therefore, the original PI is not robust enough.

For MUSIC, there may be wrong nulls in non-direction of interference. Since

$$\forall \alpha \in C^{M},\;\alpha =k_{1}e_{1}+\cdots +k_{K}e_{K}+k_{K+1}e_{K+1}\cdots +k_{M}e_{M}$$

where *k*_*i*_ (*i* = 1 ⋯ *M*) is a real number. Therefore, the directional vector **α** of any GNSS signal *s*(*t*) can be written as $\alpha =\sum_{j=1}^{M}k_{j}e_{j}$. Then

$$W_{opt}{}^{H}\alpha =(\sum_{t=1}^{M-K}p_{t}e_{K+t}{)}^{H}\sum_{j=1}^{M}k_{j}e_{j}=(\sum_{t=1}^{M-K}p_{t}e_{K+t}{)}^{H}(\sum_{j=K+1}^{M}k_{j}e_{j}).$$ (6)

If **α** meets **W**_*opt*_^*H*^**α** = 0, then there will be a wrong null in the direction of *s*(*t*).

### 3.1 A new jamming suppression criterion

In order to solve the above problems, a constraint that gains in all directions are remained unchanged can be added in the objective function. Let

$$W^{H}\alpha =1$$ (7)

where **α** is a direction vector of a certain direction. (Eq 7) means making the gain of the direction corresponding to **α** equal to 1, namely 0 dB.

Under 2-norm, for all directions, the meaning of (Eq 7) can be expressed as

$${\left\|W^{H}A-1\right\|}_{2}^{2}=0$$ (8)

where **A** is a matrix composed of all the directions’ vectors and *rank*(**A**) = *M*, **1** = [1, 1,…,1] is a row vector.

Supposing that there is an antenna array with ***M*** units, and the interval is 1 degree in both azimuths and elevations, then **A** is an M rows and 360×90 columns matrix, and **1** = [1, 1,…,1]_360×90_.

### 3.2 The new PI anti-jamming algorithm

Adding the (constraint 8) to the original (model 3), we can get a new criterion based on multi-objective optimization:

$$\{\begin{array}{c} \varepsilon (n)=\underset{W}{\text{min}}W^{H}RW+\gamma {\left\|W^{H}A-1\right\|}_{2}^{2}\\ w^{H}h=d \end{array}$$ (9)

Where *γ* > 0 is a penalty factor, which controls the constraint’s influence on the optimization course. When *γ* is bigger, the constraint requires ${\left\|W^{H}A-1\right\|}_{2}^{2}$ being closer to 0, namely gain in any direction remains unchanged.

Next, efforts will be focused to get the solution of (Eq 9). The Lagrange function of (Eq 9) is expressed as:

$$\begin{array}{l} ℜ\left(W\right)=\frac{1}{2}W^{H}RW+\gamma \|W^{H}A-1{\|}_{2}{}^{2}\text{+}\lambda (1-W^{H}h)\\ =\frac{1}{2}W^{H}RW+\lambda (1-W^{H}h)+\gamma (W^{H}AA^{H}W-1A^{H}W-W^{H}A1^{H}+32400) \end{array}$$ (10)

where *λ* is a Lagrange factor.

Let ∇ℜ (**W**) = **0**, we can get:

$$W_{\text{opt}}={\left(R+2\gamma AA^{H}\right)}^{-1}(\lambda h+2\gamma A1^{T})$$ (11)

$$\lambda =\frac{1-2\gamma h^{T}{(R+2\gamma AA^{H})}^{-1}A1^{T}}{h^{T}{(R+2\gamma AA^{H})}^{-1}h}$$ (12)

Since **AA**^*H*^ is a positive definite matrix, there is a nonsingular matrix **P** such that **AA**^*H*^ = **P**^*H*^**P**. Since (**P**^−1^)^*H*^**RP**^−1^ is still a Hermite matrix, there is a unitary matrix **U** such that **U**^*H*^[(**P**^−1^)^*H*^ **RP**^−1^]**U** = *diag*{*λ*_1_,*λ*_2_ ⋯ *λ*_*M*_}. Let **Q** = (**P**^−1^)**U**, we can get the equation

$$Q^{H}(R+2\gamma AA^{H})Q=U^{H}{\left(P^{-1}\right)}^{H}(R+2\gamma AA^{H})P^{-1}U=diag(\lambda_{1},\lambda_{2}\cdots \lambda_{M})+2\gamma I$$

where **I** is the identity matrix. Therefore the eigenvalues of **R**+*σ*^2^**AA**^*H*^ are *λ*_*i*_ + 2*γ*, *i* = 1, 2, ⋯ *M*, which are larger than the eigenvalues of **R**. It means the new method can not only reduce the width of nulls but also improve the robustness of the PI algorithm.

Nevertheless, when the GNSS system has many antennas, the solution above is complex to compute (**R**_**xx**_ + 2*γ***AA**^**H**^)^−**1**^. Therefore, the iterative method should be used.

Known from the Least Mean Square (LMS) [17],

$$W(n\text{+1)=}W\text{(}n\text{)-}\mu \nabla_{w}ℜ(n)$$ (13)

Substituting (Eq 10) into (Eq 13), we can get

$$W(\text{n}+1)=W(n)-\mu (RW(n)-\lambda h+2\gamma AA^{H}W(n)-2\gamma A1^{T})$$ (14)

Multiplying (Eq 13) by **h**^*T*^, *λ* can be obtained as:

$$\lambda =h^{T}RW(n)+2\gamma h^{T}AA^{H}W(n)+2\gamma h^{T}A1^{T}$$ (15)

An iterative formula can be derived as well and expressed as

$$W(n+1)=W(n)[I-\mu (R-hh^{T}R-2\gamma AA^{H})]+2\mu \gamma (hh^{T}A1^{T}+A1^{T})$$ (16)

### 3.3 The new MUSIC anti-jamming algorithm

In order to solve the “wrong nulls” problem of MUSIC, we just need to adjust the coefficients of the optimal weights to prevent **W**_*opt*_^*H*^**α** = 0, for the directions without jammers. In order to do this, we also use (Eq 8) as a constraint.

Let

$$f(P)=||W^{H}A-1|{|}_{2}{}^{2}=||{\left(EP\right)}^{H}A-1|{|}_{2}{}^{2}$$

where **E** = [**e**_*K*+1_ ⋯ **e**_*M*_]_*M*×(*M*−*K*)_ and **P** = [*p*_1_ ⋯ *p*_*M*−*K*_]^*T*^. Let $\frac{\partial f(P)}{\partial P}=0$, then we can get

$$P={\left(E^{H}AA^{H}E\right)}^{-1}(E^{H}AI^{H}+IA^{H}E)$$

Thus we get the vector of coefficients **P**, which can prevent **W**_*opt*_^*H*^**α** = 0.

## 4. Experiments

In the experiments, the uniform circular array is adopted, whose radius is *R*. Assuming that the phase difference from the *k-th* unit to the reference unit is *ϕ*_*k*_, and the difference between the signals received by them can be expressed as:

$$\alpha (\theta ,\;\phi ,\;\varphi_{\text{k}})=e^{-i\times 2\pi \times R\times \text{cos}\phi \times \text{cos}(\theta -\varphi_{k})}$$ (17)

where *θ* is an azimuth angle and *ϕ* is an elevation angle.

Then **A** can be written as

$$A=\left[\begin{array}{cccc} \alpha (0,0,\varphi_{1}) & \alpha (0,1,\varphi_{1}) & \begin{array}{ccc} . & . & . \end{array} & \alpha (359,90,\varphi_{1})\\ \alpha (0,0,\varphi_{2}) & \alpha (0,1,\varphi_{2}) & \begin{array}{ccc} . & . & . \end{array} & \alpha (359,90,\varphi_{2})\\ \begin{array}{c} .\\ .\\ . \end{array} & \begin{array}{c} .\\ .\\ . \end{array} & \begin{array}{ccc} . & & \\ & . & \\ & & . \end{array} & \begin{array}{c} .\\ .\\ . \end{array}\\ \alpha (0,0,\varphi_{M}) & \alpha (0,1,\varphi_{M}) & \begin{array}{ccc} . & . & . \end{array} & \alpha (359,90,\varphi_{M}) \end{array}\right]$$

In this part, both simulation and outdoor experimental testing are implemented with GPS signals of band L1.

**Fig 1:**
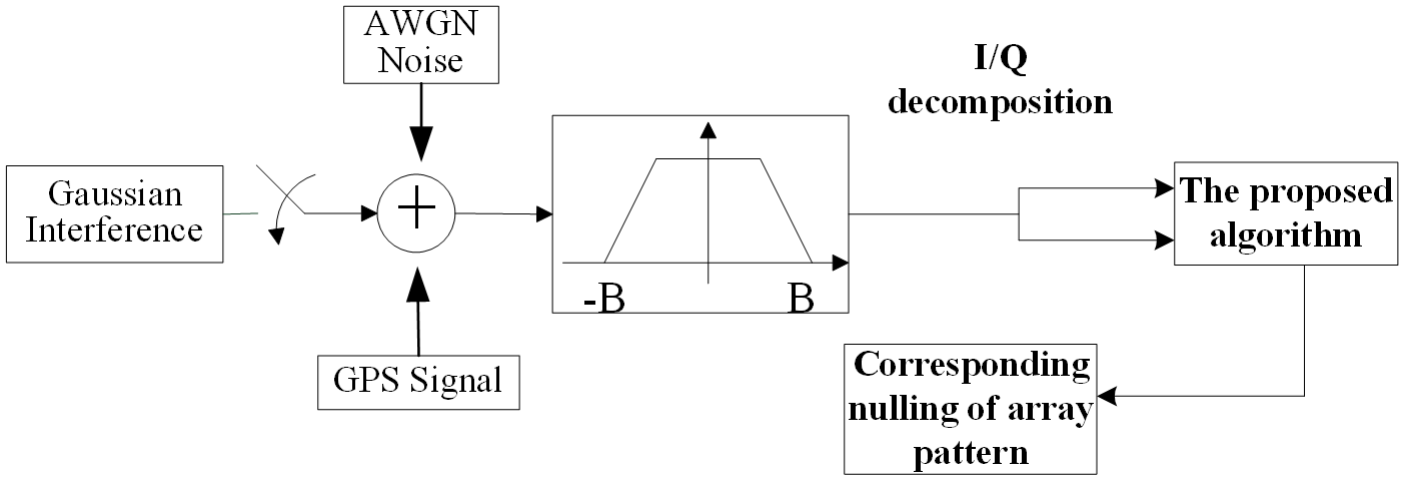
Simulation procedure of the experiments.

In the simulation experiments, the GPS signals are generated by simulation. The power of GPS signals and jammers are set to -130 dBm and -70 dBm respectively. The jammers are 1500km away from the antenna array. The simulation procedure is shown in Fig 1.

### 4.1 Simulation and analysis of the new PI algorithm

**Fig 2:**
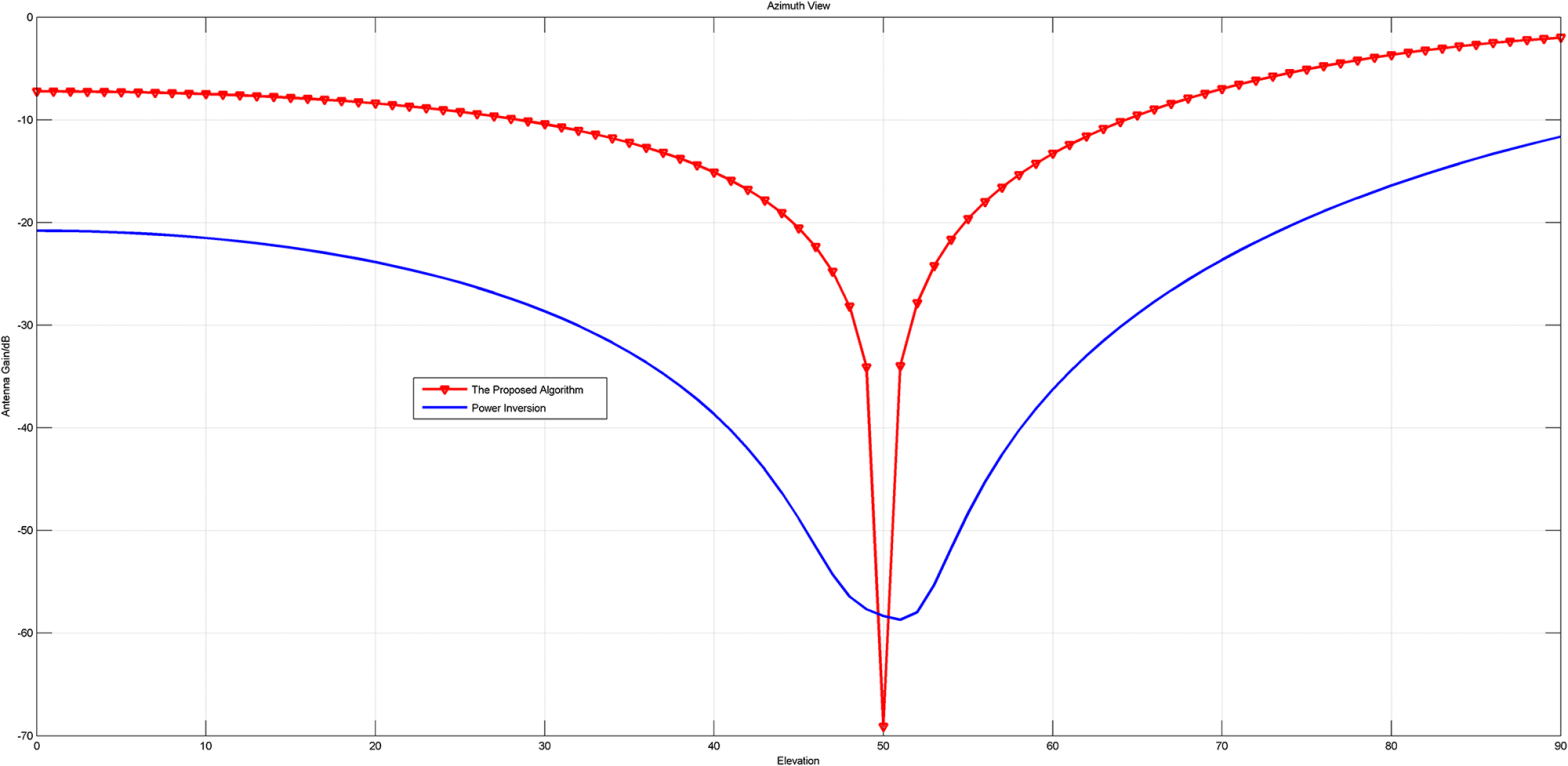
The pattern in azimuth view of the array patterns of the original PI and the proposed PI with a narrow band jammer.

**Fig 3:**
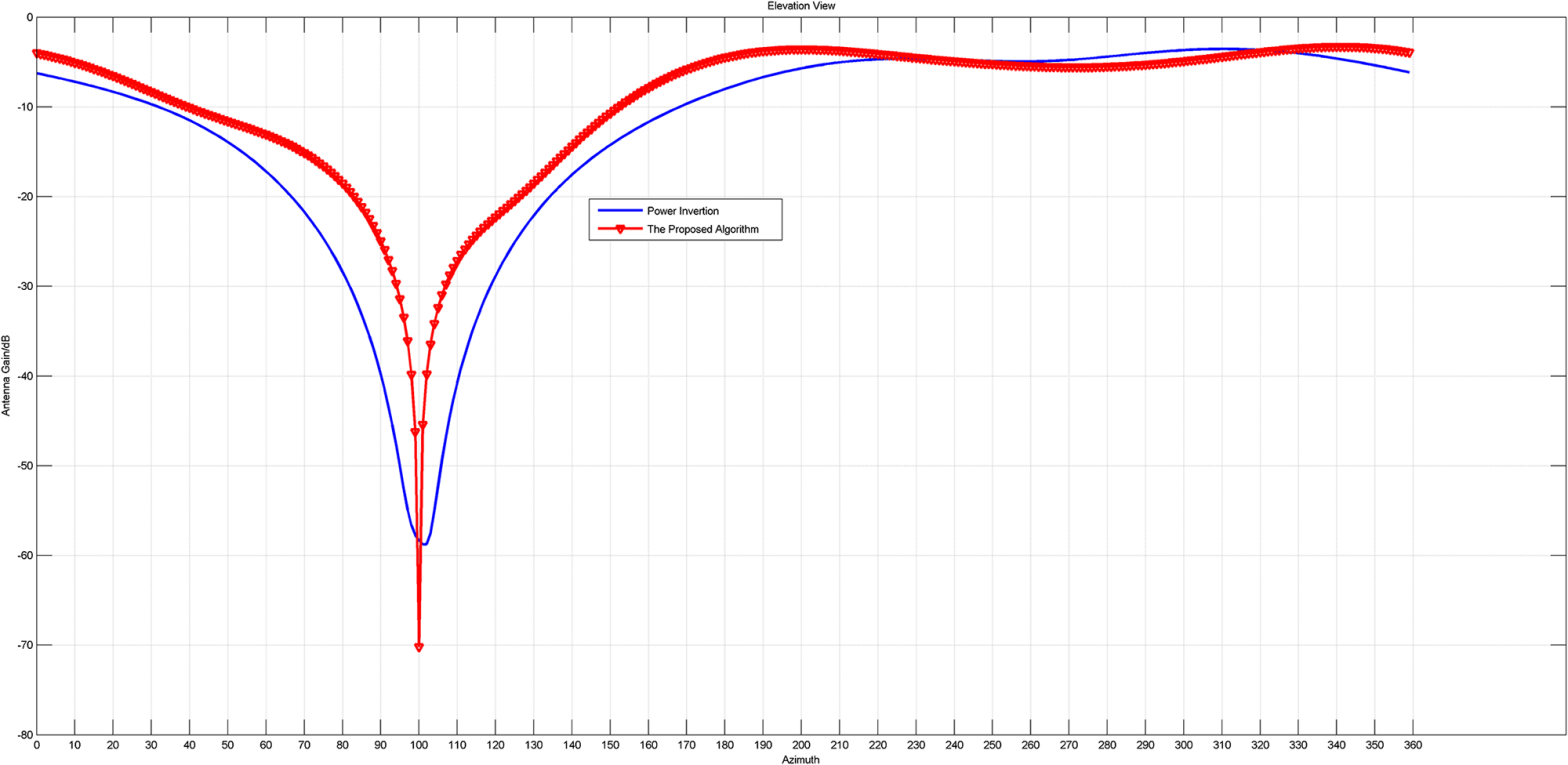
The pattern in elevation view of the array patterns of the original PI and the proposed PI with a narrow band jammer.

In this experiment, the array with 4 units is adopted. Firstly, the narrowband signal is chosen as the jammer, whose direction is *θ* = 300°, *φ* = 80°. The array patterns of the original PI algorithm and the new proposed PI algorithm based on 2-norm are shown in Figs 2 and 3.

**Fig 4:**
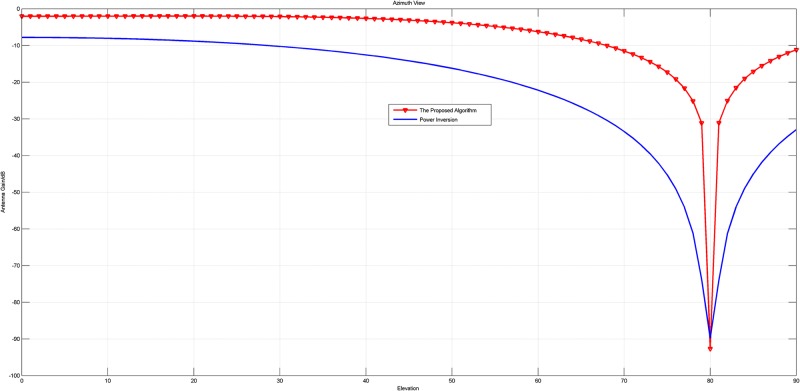
The pattern in azimuth view of the array patterns of the original PI and the proposed PI with a wide- band jammer.

**Fig 5:**
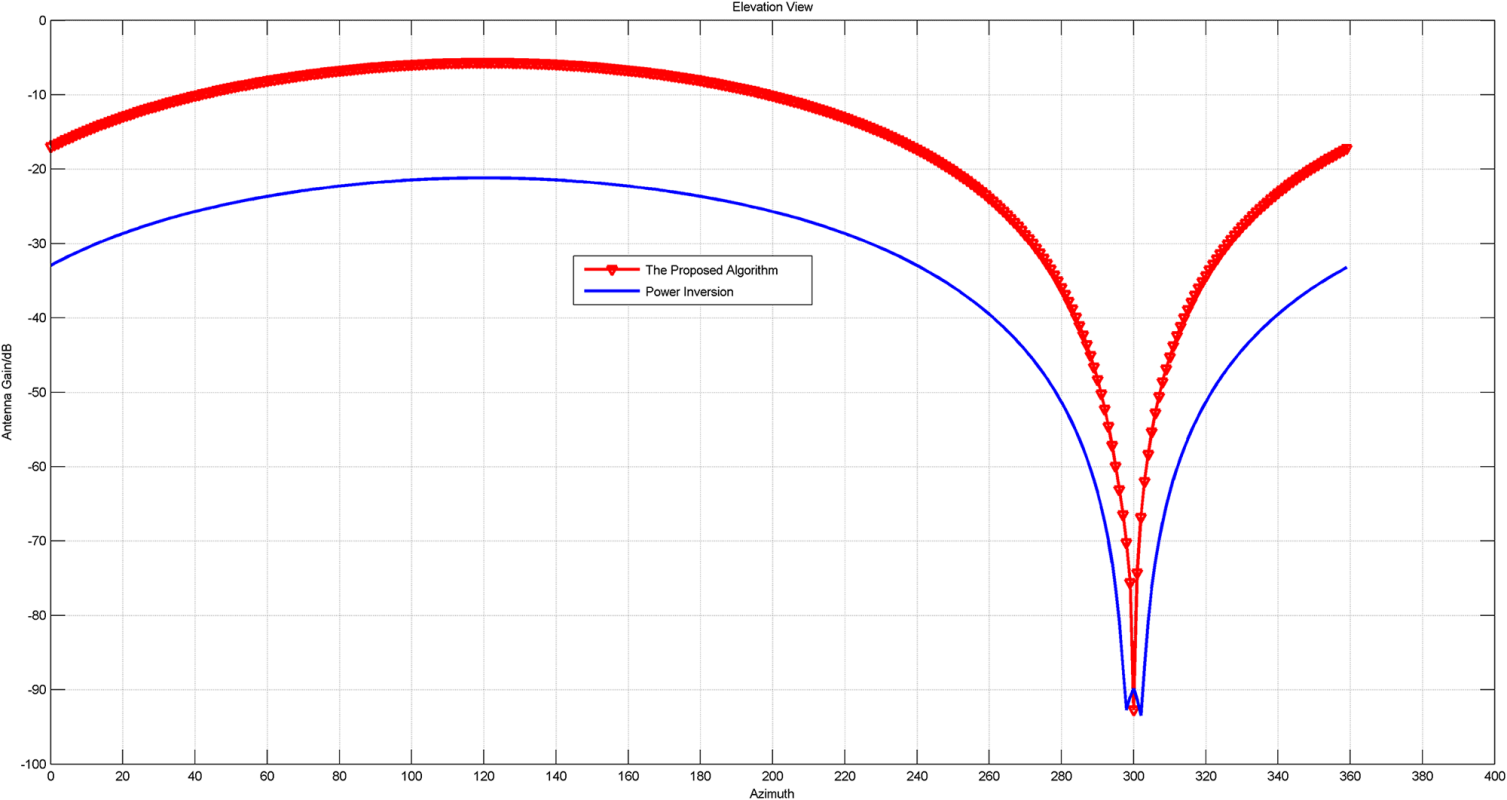
(a) The pattern in elevation view of the array patterns of the original PI and the proposed PI with a wide- band jammer.

When the jammers change to Pulse Modulation signals (a wideband jammer), whose direction is *θ* = 100°, *φ* = 50°, the corresponding patterns are displayed in Figs 4 and 5.

Figs 2 to 5 intuitively reveal that the array patterns of the proposed algorithm is narrower in nulls and more flat in non-interference direction than the original PI algorithm no matter the jammer is narrowband or wideband.

### 4.2 Simulation and analysis of the new MUSIC algorithm

In this experiment, the array with 6 units is applied. There are four jammers, which are a sweep-frequency signal with *θ* = 120°, *φ* = 50°, a CW(Continuous Wave) signal with *θ* = 200°, *φ* = 40°, a frequency modulated signal with *θ* = 60°, *φ* = 70°, and a white Gaussian noise signal with *θ* = 100°, *φ* = 20°.

**Fig 6:**
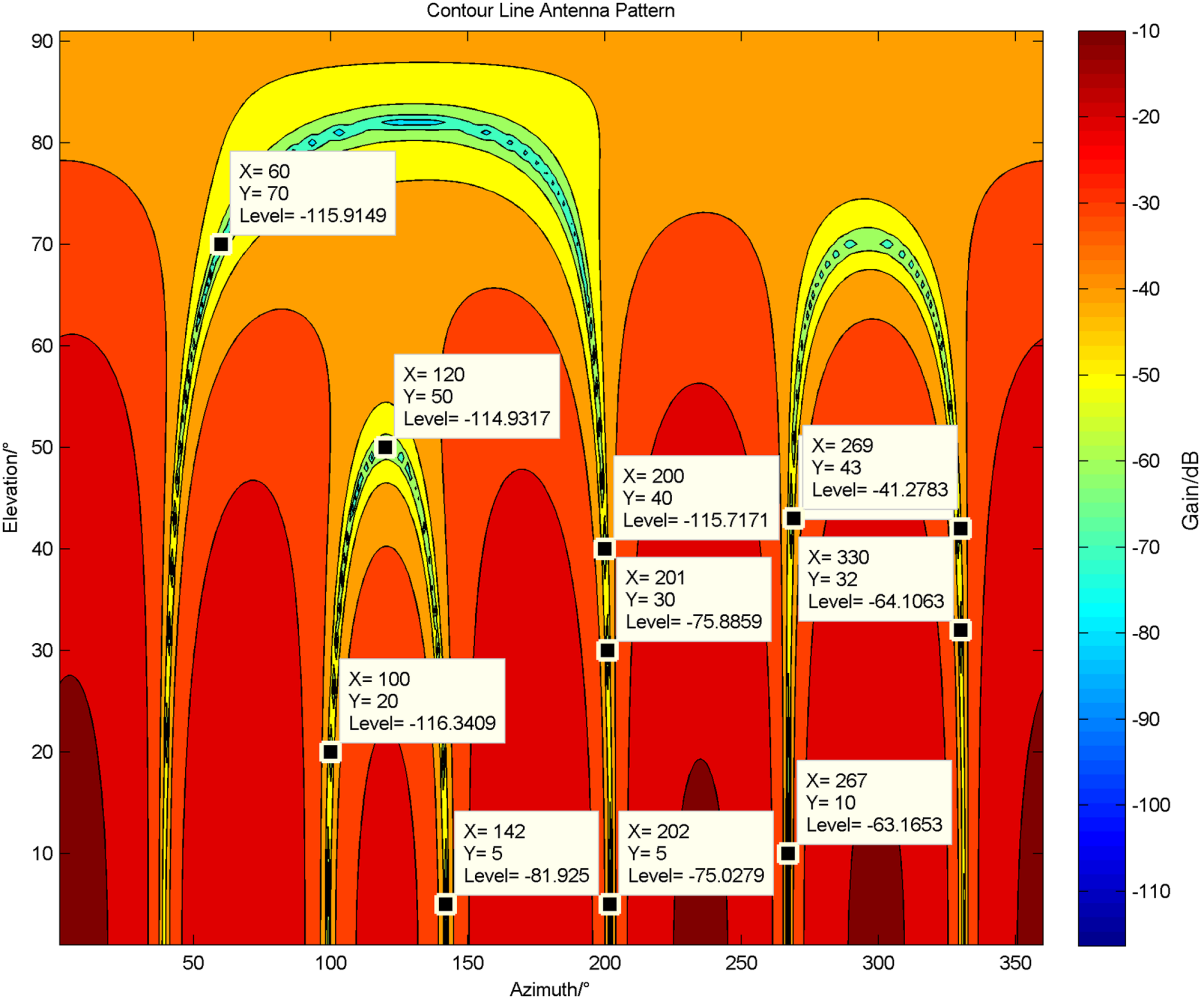
The 2-dimensional array pattern by using contour lines after the adaptive processing of the original MUSIC algorithm.

**Fig 7:**
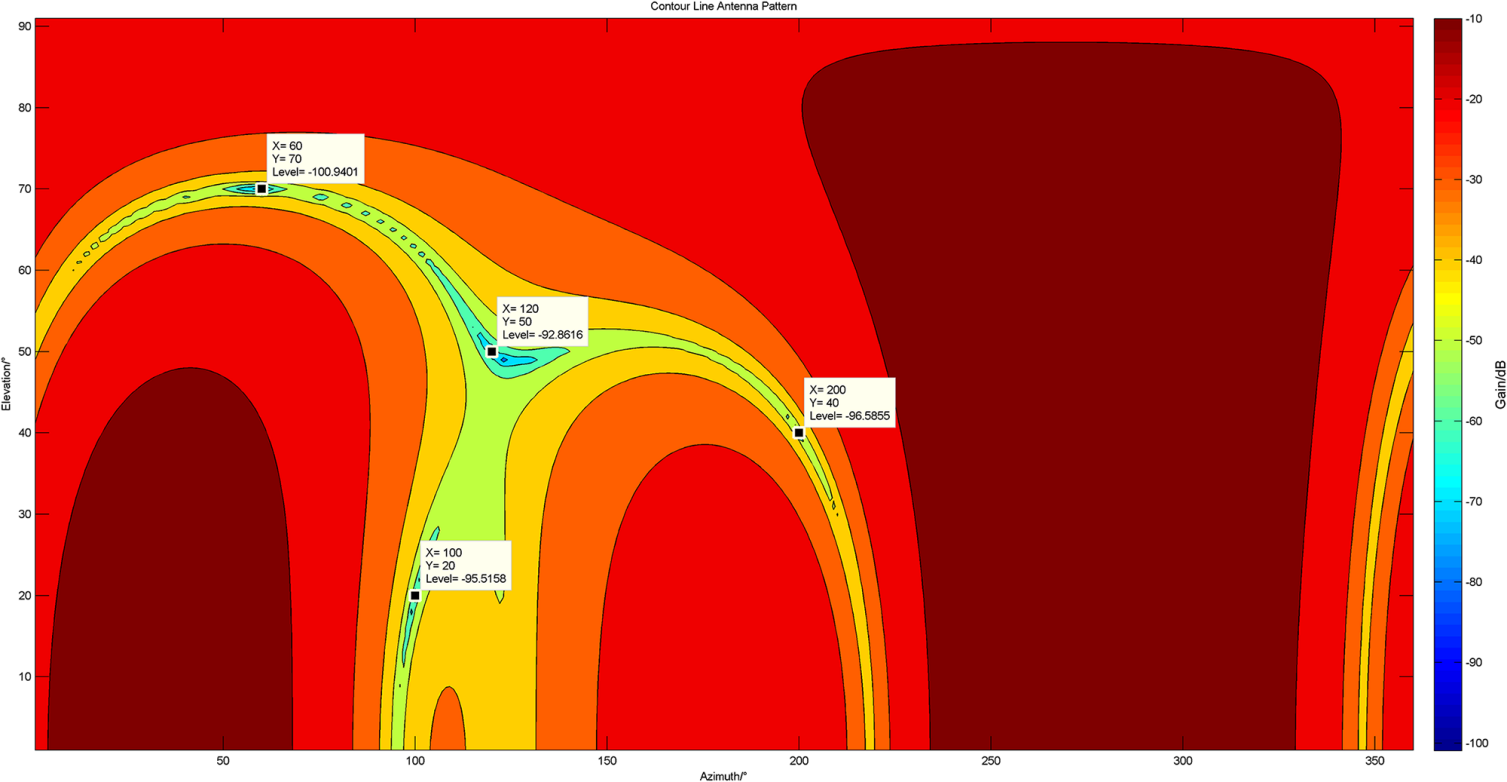
The 2-dimensional array pattern by using contour lines after the adaptive processing of the proposed 2-norm matrix constrained MUSIC algorithm.

In Fig 6, we set all *p*_*i*_ (*i* = 1 ⋯ *M* − *K*) to be 1, and show the 2-dimensional array pattern by using contour lines. In comparison, under the same condition, the proposed algorithm is employed to suppress the jammers and its corresponding array pattern is shown in Fig 7.

Fig 6 obviously shows that the original MUSIC algorithm forms deep notches at the exact locations where jammers exist, whereas wrong nulls also appear in the space where there is no interference at all, such as the locations marked in Fig 6. Once the GPS signals are located there, they will be filtered so that the receivers cannot capture them, which could greatly influence the performance of GPS receivers.

However, in Fig 7, it is fortunate that the undesired notches in Fig 6 vanish and the needed notches emerge exactly over there. This is absolutely beneficial to the receivers. Moreover, it is helpful to distinguish the spectrum peak more quickly when the proposed algorithm is applied to DOA estimation.

### 4.3 Outdoor experiments and analysis

The proposed algorithm is also verified with practical outdoor experiments. The anti-jamming experiments were done in an open, empty and non-blocked area.

**Fig 8:**
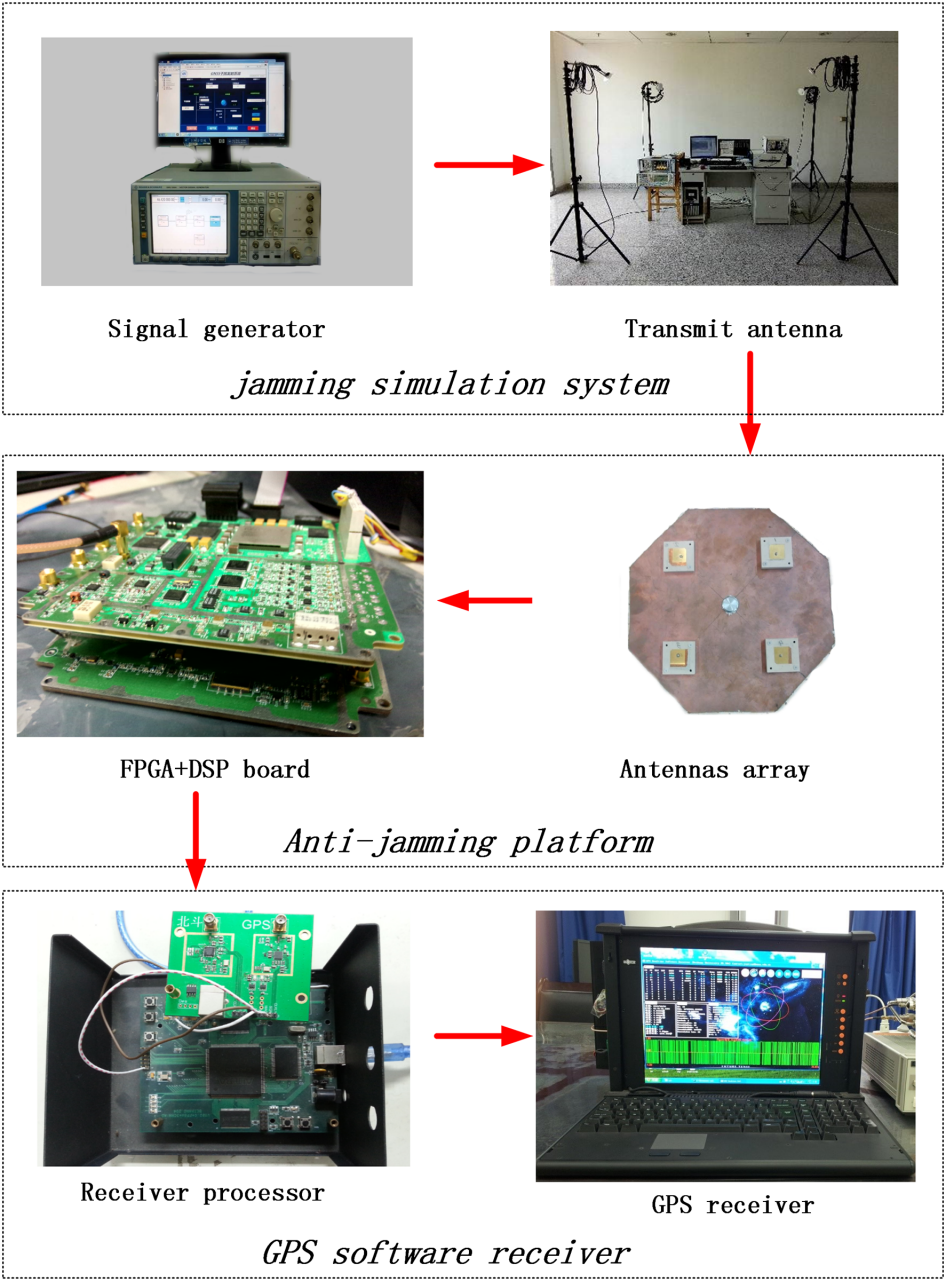
The experiment setup.

In this experiment, the testing setup is composed of three parts, including jamming simulation system, anti-jamming platform and the GPS software receiver, as demonstrated in Fig 8. The jamming simulation system consists of a signal generator and four transmitting antennas. The anti-jamming platform is made up of a FPGA (Field Programmable Gate Array) circuit board, a DSP (Digital Signal Processing) board and a 4 elements uniform circular array. The GPS software receiver has a GPS receiver and the corresponding processing parts. The jammer emits different kinds of jamming signals. The proposed anti-jamming algorithm is written into the FPGA board and its interface is controlled by a DSP board. The results from the FPGA are transmitted to the GPS software receiver.

In the outdoor tests, the jamming signals are narrowband Continuous Wave (CW) and wideband Gauss noise with bandwidth being 2.046MHz. Under the same jammer-to-signal ratio (JSR), the *C*/*N*_0_ (carrier-to-noise) of the same satellite is compared, when the platform utilizes the original algorithms and the proposed new algorithms respectively. It should be emphasized that *C*/*N*_0_ ratio is a key index for the receiver to capture and track the satellites. In our receiver, we set the threshold *C*/*N*_0_ ≥ 38dB as the capturing requirement. The results of comparing the algorithms under the same satellite are displayed in Table 1.

**Table 1:** The experimental results of single satellite comparison.

Jammer type /JSR	Carrier-to-noise after antijamming by the original PI	Carrier-to-noise after antijamming by the proposed PI
CW(narrowband)/ JSR = 65dB	48.2 dB	50.1 dB
CW(narrowband)/ JSR = 74dB	44.6 dB	47.3 dB
CW(narrowband)/ JSR = 82dB	40.7 dB	43.2 dB
Gauss-noise(wideband)/ JSR = 55dB	40.1 dB	42.8 dB
Gauss-noise(wideband) /JSR = 60dB	39.2 dB	41.7 dB
Gauss-noise(wideband)/ JSR = 65dB	non-tracked	38.2 dB

Theoretically, the receiver is able to provide position service if four or more satellites are captured and tracked. The satellites with *C*/*N*_0_ ≥ 38dB and the correlation value satisfying a certain threshold are defined as tracked satellites. As for positioning satellites, they are strictly selected from the tracked satellites whose navigation messages can be well calculated and are less disrupted [18]. Usually the more the positioning satellites are, the more precise the position results will be. For further testing, we compare the performance of the algorithms under their corresponding maximum JSR. The maximum JSR is the maximum anti-interference ability of the algorithm, which means after anti-jamming by the algorithm the receiver is still able to position. The results are filled in Table 2.

**Table 2:** The experimental results of multiple satellites comparison.

Algorithms/Jammer type	Maximum JSR	Positioning satellites/Tracked satellites	Positioning error
The proposed PI algorithm/CW signals	JSR = 85dB	6/9	≤ 10 meters
The original PI algorithm/CW signals	JSR = 83dB	4/7	≤ 90 meters
The proposed PI algorithm/Gauss-noise	JSR = 63dB	6/8	≤ 10 meters
The original PI algorithm/Gauss-noise	JSR = 63dB	4/7	≤ 150 meters

From Table 1, it can be found that as far as the *C*/*N*_0_ is concerned, the proposed algorithm behaves better than the original one no matter the jammers are narrowband or wideband. This is significant since higher *C*/*N*_0_ ratio means more tracked satellites. From Table 2, it also can be seen that the proposed algorithms have more positioning satellites then original algorithm under the same interference intensity. Usually more positioning satellites will be more beneficial to the positioning accuracy. This is reflected in Table 2.

Obviously from the entire experiments, the proposed algorithm has better interference suppression ability and can improve the positioning capability of the receivers.

## 5. Conclusions

A multi-objective optimization jamming mitigation algorithm with 2-norm is proposed in this paper, in order to solve the problem that nulls corresponding to jammers are too wide and the wrong nulls problems in the spatial adaptive jamming suppression algorithms. The direct inverse method and an improved iterative PI algorithm with multi-objective optimization based on 2-norm have been presented. The new MUSIC is also present to avoiding the wrong nulls. Both simulation and practical outdoor experiments are carried out on the basis of GPS signals in band L1 to verify our proposed idea. The corresponding simulation and testing results show that the dedicated algorithms with multi-objective optimization based on 2-norm can make the nulls narrower and the gains in the non-jammed directions more flat. Moreover it raises the entire gains of the arrays, and achieves promotion of GNSS signals’ SNR.

## Supporting information

S1 Dataset S1_Data is the data with the interference in (100,50). (MAT)

S2 Dataset S2_Data is the data with the interference in (300,80). (MAT)
